# Changes in the Oral Microbiota with the Use of Aligners vs. Braces: A Systematic Review

**DOI:** 10.3390/jcm13237435

**Published:** 2024-12-06

**Authors:** Pilar España-Pamplona, Laura Bernés-Martínez, Carolina Andrés-Castelló, Belén Bolás-Colveé, Milagros Adobes-Martín, Daniele Garcovich

**Affiliations:** Department of Dentistry, Universidad Europea de Valencia, Paseo de la Alameda 7, 46010 Valencia, Spain

**Keywords:** orthodontic aligners, oral microbiota, systematic review, dental braces, clear aligners, periodontal health, microbial changes, oral hygiene, microbiome dynamics

## Abstract

**Background:** Orthodontic treatments have evolved significantly, with clear aligners becoming increasingly popular due to their aesthetic appeal and ease of use. This study systematically reviewed the impact of clear aligners in the changes in the oral microbiota compared to traditional fixed appliances. **Methods:** Following PRISMA guidelines, a systematic review was conducted using two databases such as Scopus, Web of Science, and the PubMed search engine. The studies included were those published between 2010 and 2023, involving adults over 18 years using clear aligners and fixed appliances. The data on oral microbiota changes were extracted and analyzed. **Results:** The review included eight studies, highlighting the differences in microbial changes associated with clear aligners versus fixed appliances. Clear aligners were associated with fewer detrimental changes in the oral microbiota, potentially due to their removable nature allowing for better hygiene. Fixed appliances showed a tendency to harbor more pathogenic bacteria, which is likely due to their difficulty to clean. **Conclusions:** Clear aligners may offer a better alternative to fixed appliances in terms of maintaining a healthier oral microbiota. Their design and ease of hygiene contribute to less accumulation of pathogenic bacteria, showing a more positive impact on maintaining a balanced oral microbiota when compared to fixed appliances.

## 1. Introduction

The journey of orthodontics from its simple beginnings, marked by Christophe François Delabarre’s invention of the wire crib in 1819 [1,2] to today’s sophisticated technologies, illustrates a fascinating evolution. These advancements have dramatically transformed the approach to dental care, focusing not just on aesthetics but also on improving oral health and functionality [1,2,3,4].

At the end of the 20th century, clear aligners were widely introduced, marking a major shift from the traditional, often uncomfortable, braces to more aesthetic and less noticeable solutions [5]. The integration of computer-aided design and manufacturing (CAD/CAM) further enhanced the functionality and aesthetic appeal of these devices, allowing for the precise movement of teeth, and tailored treatments to individual needs [2,5,6,7].

Today, the focus in orthodontics is significantly on enhancing patient comfort and aesthetic appeal. Clear aligners are at the forefront, offering an almost invisible, removable, and custom-fit alternative to traditional braces [8]. These aligners provide significant advantages, such as the ability to remove them for eating and cleaning, appealing to a broader demographic including teenagers and adults [9]. But the introduction of clear aligners has raised questions about their impact on the oral microbiome. Unlike traditional braces, which can trap food particles and bacteria, leading to dental issues like caries and periodontal disease, clear aligners are designed to be smooth and removable, potentially reducing these risks by promoting better oral hygiene [10,11].

The oral microbiome is a complex ecosystem, hosting over 1000 known bacterial taxa, along with fungi, viruses, and protozoa. A variety of methods have been developed and applied for the detection and identification of microorganisms. Bacterial culture has long been considered the gold standard, although these techniques are extremely delicate and require experienced staff and strict quality assurance procedures [12].

These organisms are crucial for maintaining both oral and systemic health [11,12,13,14]. Their ability to form biofilms is particularly significant as these structures contribute to the development of dental conditions such as caries and periodontitis. Additionally, the balance of these microbial communities is vital, as dysbiosis is linked to various systemic diseases, underscoring the profound impact of oral health on overall well-being [13].

For all this reasons, the overarching goal of this research was to investigate the impact of clear and fixed aligners on the oral microbiota because of the importance of maintaining an adequate oral microbiota in the conservation of oral and general health.

## 2. Materials and Methods

This systematic review was conducted following the preferred reporting items for systematic reviews and meta-analyses (PRISMA) Guidelines for 2020. These guidelines provided a comprehensive framework for planning, executing, and reporting on the systematic review process. The review protocol was registered in the Open Science Framework ((OFS), https://osf.io/jurxe/ accessed on 24 September 2024), but it is not publicly accessible.

The search strategy and process were performed following the scheme of the following PICO question for this systematic review: “(P) In orthodontics patients (I) treated with clear aligners (C) or fixed appliances (O) is there a difference related to the changes in the oral microbiota with variables such as Plaque Index (PI), Gingival Index (GI), Bleeding on Probing (BI), Probing Depth (PD) and the amount of anaerobic bacteria after treatment?” The null hypothesis was that there were no differences among these indicators and the modality of orthodontic treatment.

### 2.1. Eligibility Criteria

The inclusion and exclusion criteria were developed in accordance with the previously reported PICO question. The included studies were as follows: controlled clinical trials (CCTs), where a number of similar people were randomly assigned to 2 (or more) groups; randomized clinical trials (RCTs); cohort studies and observational studies both, retrospective and prospective; published or in press; published from 2010 to 2024; published in English; comparing the variables included in the PICO questions in patients treated with clear aligners or conventional fixed appliances. The exclusion criteria were as follows: reviews; case reports; case series (less than 10 cases); animal studies; clinical studies not comparing the two treatment modalities; incomplete text.

### 2.2. Information Sources

An electronic search was performed on the following databases: PubMed, Scopus, and Web of Science (Science Citation Index Expanded). The search was conducted on 31 March 2024. The time filters were applied from 2010 to 2024. A manual search of additional articles related to the topic was performed using the references of the selected articles.

### 2.3. Search Strategy

The search strategy was developed to include specific terms related to clear aligners, fixed appliances, and the microbiota, employing Boolean operators to refine the search results. Due to the differences in regulated vocabulary and syntax restrictions, detailed search algorithms were divided and suitably applied for each database, but the foundations of the search, including the words and Boolean search terms, was the same (Table 1).

### 2.4. Study Selection

The selection process was carried out by two researchers (P.E.P., L.B.M.) according to the criteria of the PRISMA protocol [15]. The authors were calibrated first and presented a high inter-operator reliability (Kappa = 0.87). To measure interrater reliability, two researchers conducted the same observation on the same sample. Then, we calculated the correlation between their different sets of results. The retrieved articles were first selected by titles and abstracts, selecting any potentially eligible studies. Subsequently, the same researchers performed a second screening of the full text of the articles according to the defined inclusion and exclusion criteria (type of study, no significant results, and other languages). The screening involved a two-phase process using Zotero 7^®^ software (VA, USA) to manage and remove duplicate entries. Data extraction focused on key variables relevant to the study objectives, including the type of orthodontic appliance used (clear or fixed), the age of the patients, the methods of microbiota analysis, and the outcomes related to oral health indices such as plaque and bleeding indices. In any cases of disagreement, a third reviewer was consulted (C.A.C.).

### 2.5. Data Collection Process

The data of the selected articles were extracted by one reviewer and exported to an Excel datasheet (Microsoft Office for Mac 2011 package, WA, USA), organized according to the Cochrane Consumers and Communication Review Group’s data extraction template. A second author checked the extracted data and disagreements were resolved by consensus. In this phase, no author was contacted, since all numerical data were provided in the published papers.

### 2.6. Data Items

The following data were extracted: (a) name of the authors; (b) year of publication; (c) study type; (d) sample size of the group treated with FA; (e) sample size of the group treated with CA; (f) sex of the participants; (g) average age and SD; (h) treatment/observation time; (i) outcome variables analyzed; (j) conclusions. Unless the same data were gathered from at least two of the selected studies, the relevant data could only be described but not synthesized. The following outcome variables for periodontal health assessment were considered: plaque index (PI), probing depth (PD), bleeding on probing (BOP) and microbial changes.

### 2.7. Quality Assessment

An independent quality assessment of the included studies was performed by two reviewers (P.E.P, L.B.M.) and any disagreement was discussed and resolved by consensus when necessary. The Newcastle–Ottawa scale (NOS) was used for assessing the quality of non-randomized studies, including cohort and case-control studies. The NOS scores facilitated the conducting of sensitivity analyses to explore the impact of study quality on the overall results of the review, comparing findings across studies of varying quality [16]. (Table S1). The quality assessment for RCTs was performed using the Cochrane risk of bias 1 tool (RoB1) [17]. The tool assesses the following items: (1) random sequence generation; (2) allocation concealment; (3) blinding of participants and personnel; (4) blinding of outcome assessment; (5) incomplete outcome data; (6) selective reporting bias; (7) other bias. Every single item can be considered as having a low risk, being unclear, having a high risk or having no information (Figure S1).

## 3. Results

This systematic review identified 185 articles across three platforms: PubMed (72), Scopus (62), and Web of Science (51). After removing duplicates, 140 articles were screened for relevance. The initial screening led to the exclusion of 103 articles. Further assessment for eligibility was conducted on 37 articles, of which 10 were not retrievable due to the lack of accessible full-text versions. An additional 19 articles were excluded for not meeting the study criteria, leaving 8 studies to be included in the systematic review. The PRISMA flow chart (Figure S2) visually summarizes the selection process.

This systematic review included eight studies, rigorously selected to cover a range of designs and objectives. Four studies focused on the effects of various orthodontic appliances (both fixed and clear aligners) on oral microbiota and periodontal health. Three studies assessed periodontal parameters to compare different orthodontic treatments, and one study monitored periodontal health over time in patients using fixed orthodontic appliances. The studies encompassed 531 participants across continents including Africa, Asia, North America, and Europe, with ages ranging from 18 to 50 years [18,19,20,21,22,23,24,25]. One study included younger participants aged 16 to 21 [19]. Exclusion criteria typically included lifestyle factors like smoking, systemic diseases affecting oral health, current medication use impacting oral or gingival health, and pregnancy. The orthodontic interventions examined were metal brackets, ceramic brackets, self-ligating brackets, and clear aligners [18,19,20,21,22,23,24,25].

### 3.1. Effects on Microbial Changes

Key measurement tools included periodontal health indices, such as plaque indices, bleeding indices, and pocket depth, along with PCR for microbiological evaluation. As microbiological assessments, studies like Sanz-Orrio-Soler et al. [18] used saliva swabs (Ref. 551291 in Francisco Soria Melguizo S.A. (Spain)) and Sabouraud Dextrose Agar plates to measure Candida albicans colonization pre- and post-treatment with fixed appliances.

Table 2 highlights in detail how orthodontic appliances affected the oral microbiome across diverse patient demographics and methodologies. Two studies [21,22] noted worsening oral health and elevated Streptococcus mutans respectively, indicating increased risks with long-term orthodontic treatment. Babikow (2023) [23] observed significant shifts towards disease-associated microbial species. Conversely, Sfondrini [24] and Sanz-Orrio-Soler [18] found negligible changes in microbial levels with aligner therapy, while Levrini [19] recommended clear aligners for better periodontal outcomes. Mulla Issa [20] emphasized clear aligners’ superiority in maintaining periodontal health compared to other braces.

### 3.2. Effects on Periodontal Indices

Levrini et al. [19] employed PCR to assess periodontopathogen bacteria in patients with fixed appliances compared to those with clear aligners. The plaque index dropped from 0.39 to 0.30 for patients wearing transparent aligners over the course of two months, whereas the probing depth stayed constant at about 1.6 mm. While not statistically significant, bleeding on probing dropped somewhat, from 4.55 to 4.08. In comparison to patients with transparent aligners, patients with fixed appliances showed a higher plaque index, a steady probing depth of around 1.3 mm, and a notable rise in bleeding on probing over time.

As clinical periodontal assessments, Mulla Issa et al. [20] and Vrakova et al. [21] used indices such as plaque index (PI), gingival index (GI), and bleeding on probing (BI) to evaluate the impact of different orthodontic appliances on periodontal health. For a specific pathogen load, researchers included PCR assays to detail the influence of various appliances on periodontopathic bacteria and general microbial shifts due to hygiene practices associated with different orthodontic appliances [20,23,24,25].

The impact on the periodontal health indices is summarized in Table 2. Levrini et al. [19] and Mulla Issa et al. [20] reported that clear aligners significantly reduce the plaque index (PI) and improve bleeding on probing (BOP), while maintaining stable probing depth (PD). In contrast, fixed appliances showed generally poorer outcomes. Clear aligners were associated with a reduced PI and a stable PD and BOP, reflecting decreased microbial risks. Fixed appliances, however, showed negative impacts, such as an increased PI and worse periodontal health, particularly in less accessible areas. Studies noted that clear aligners maintained a healthier microbiome and better periodontal indices than fixed appliances, which showed no significant changes in PD, BOP, or PI but an increase in anaerobic bacteria post-treatment, indicating a shift towards dysbiosis. Clear aligners tended to maintain a healthier and less pathogenic microbiome than fixed appliances. They inhibited growth of periodontitis-associated pathogens and supported better periodontal health [18,19,20,21,22,23,24,25]. Conversely, fixed appliances increased pathogenic bacteria levels and complicated oral hygiene maintenance, leading to poorer periodontal outcomes [21,22,23,24,25].

## 4. Discussion

This systematic review analyzed studies with diverse designs, including controlled trials and cross-sectional studies. Each study was assessed for bias using NOS indices [16] or the Cochrane risk of bias 1 tool (RoB1) [17] in the case of the RTCs. The highest level of scientific evidence was observed in the RTC of Levrini et al. [19], which supported the hypothesis that clear aligners significantly benefit oral microbiota health compared to fixed appliances. Despite the robust methodology, limitations such as the relatively recent popularity of clear aligners and the absence of large-scale long-term studies were noted [22,23,24,25].

Sampling techniques across the studies typically involved non-invasive methods like saliva swabs, which are favorable for their ease and consistency in sample collection [18,19,20,21,22,23,24,25]. This approach, however, is susceptible to variability based on the collection site and timing. To minimize the risk of heterogeneity in the results, a previous systematic review suggested the standardization of collection times, particularly before any oral hygiene activity, to maintain the integrity of the microbiota sample [26].

Another issue to consider would be the hygiene protocol carried out by each study, as this could directly influence changes in the oral microbiota. As with other variables, the studies analyzed did not present homogeneity in the method used. The study of Sfondrini et al. [24] used a very complete cleansing protocol consisted in a professional supragingival and subgingival oral hygiene using a piezoelectric and Gracey curettes (Hu-Friedy, Chicago, IL, USA) with a final periodontal pockets decontamination by means of Air-Flow Plus (EMS SA). Participants were then instructed to follow a correct oral hygiene routine at home consisting of the use of an electric toothbrush for 2 min three times a day, as well as of the use of a dental floss once a day, abstaining from mouthwash to avoid an alteration of the resident bacterial flora. A similar protocol was used in the study of Levrini et al. [19], where patients used no oral antiseptic solutions or mouthwash during the entire investigation. Oral hygiene instructions were specified by an experienced dental hygienist before the treatment and recapitulated during all the scheduled check-ups. By contrast, electric toothbrushes were not allowed. All patients had to use an orthodontic brush (bass technique for 2 min) and dental floss three times a day. In the study of Jing et al. [22], all subjects were required to use fluoride-containing toothpaste according to oral hygiene instructions, but in the study of Mulla Isa et al. [20], they excluded patients who used antiseptic solutions or mouthwash during the past 6 months or underwent any periodontal treatments in the past 6 months. According to their knowledge, they were the first study to use the basic periodontal examination index (BPE index) to assess periodontal health in orthodontics patients. No cleansing protocol was used in the study of Babikow et al. [23], as well as in other studies [18,21,25]. In the case of Sanz-Orrio-Soler et al. [18], patients completed a questionnaire related to their hygiene habits. The results of this initial questionnaire on volunteer’s oral hygiene were cross-referenced with the presence or absence of Candida in the oral cavity during the study. Similarly, in the study of Marinkak Vrankova et al. [21], patients filled in the questionnaire including questions about smoking, alcohol, diet, taste preferences, and oral hygiene. The overall status of oral hygiene (classified as excellent, good, or poor oral hygiene) was also clinically evaluated.

The studies employed a range of analytical techniques to assess the oral microbiota, including PCR, qPCR, and next-generation sequencing [21,22,23,24,25]. These methodologies provided a comprehensive view of the microbiota dynamics, indicating that clear aligners tended to maintain a healthier oral environment, likely due to less disruption of natural cleaning behaviors and better overall hygiene accessibility [18,19,20,21,22,23,24,25].

Upon careful analysis of the aligners used in the studies included in this systematic review, Levrini et al. [19] and Mulla Issa et al. [20] examined Invisalign aligners (USA), though the latter sample also included patients who used Angel Aligners (China). Sfondrini et al. [24] used Arc Angel Aligners (Modena, Italy). Unclean aligners can harbor harmful bacteria and plaque, and plaque can cling to the plastic aligners in the same way that it does to teeth [19]. Despite this, the results consistently indicated that clear aligners were more effective in maintaining a healthier oral microbiota than fixed appliances. Studies highlighted the reduced prevalence of periodontopathogen bacteria and an overall improvement in oral health markers, such as plaque indexes and periodontal indices, in patients using clear aligners [18,19,20,24,25]. At the periodontal level, CA patients presented better indicators than FA patients; the plaque index, bleeding index, gingival index, and probing depth were significantly worse in the FA group. According to a meta-analysis found in the literature, clear aligners were shown to be effective not only in orthodontic treatment but also in promoting a healthier oral microbiota [27]. As in our findings, this was attributed to their design, which facilitates easier cleaning and less bacterial accumulation compared to more traditional, fixed orthodontic appliances [18,19,20,21,22,23,24,25,28]. The studies included in this research suggested that, due to the use of no or fewer screws, the plastic materials, and less crevices, clear aligners were less conducive to bacterial growth, which helps in reducing overall microbial load. However, appliances that are fixed, due to their materials and the use of braces and wires, provided more surface area for bacteria to adhere to and prone themselves therefore to more bacterial growth. Compared to fixed appliances, clear aligners consistently demonstrated a positive impact on oral hygiene and microbiota health. The studies reviewed provided evidence of lower bacterial loads and improved periodontal health in patients using clear aligners [18,19,20,21,22,23,24,25].

It seems necessary to mention occlusal trauma as a reason for orthodontic treatment need. According to a study found in the literature [29], secondary occlusal trauma associated with periodontal disease is characterized by significantly higher RANKL levels in patients with chronic occlusal trauma. This partially clarifies the molecular mechanisms that underlie more severe tissue destruction in patients with occlusal trauma. RANKL is the abbreviation for the receptor activator of the nuclear factor kappa beta. It is an apoptosis regulator gene, a binding partner of osteoprotegerin (OPG), a ligand for the receptor RANK, and it controls cell proliferation [30]. Due to the relationship between the modality of orthodontic treatment and oral microbiota found in this systematic review, where reduced prevalence of periodontopathogenic bacteria and an overall improvement in oral health markers, such as plaque indexes and periodontal indices in patients using clear aligners, were provided [18,19,20,24,25], more studies should be carried out in the future to understand the effect of our orthodontics treatments in the oral microbiota and their consequences.

We found other systematic reviews in the literature that have addressed this issue in the past [31,32,33,34]. As in our study, Oikonomou et al. [31] found that patients treated with aligners and no additional attachments/adjuncts presented potentially higher levels of oral health overall. For periodontal parameters, adults undergoing aligner orthodontic treatment presented summary plaque scores that were 0.58 lower than those treated with fixed appliances, while no evidence of difference was recorded for inflammation indices. However, the evidence was supported by low to very low certainty.

As pointed out by Chavaret et al. [28], although removable orthodontic clear aligner appliances can be removed for eating, drinking, and performing oral hygiene routines, the adhesion and growth of bacterial biofilms on these appliances is possible. Therefore, this problem should not be ignored. Rouzi et al. [32] concluded that the removable nature of clear aligners makes oral hygiene procedures easier and more effective for orthodontic patients. Patients must use a multistep cleaning and disinfection technique that combines mechanical and chemical methods in order to keep aligners clean to prevent biofilm infection.

ElNaghy et al. [33] found that there was a tendency to report better GI values with clear aligners. However, no statistical significance between the two treatment modalities was shown for any follow-up intervals. Following the same trend, periodontal indicators showed significantly better values in patients treated with clear aligners, with moderate- to large-sized effects. PI and GI had a significant tendency to improve over time. In microbiological indicators, CAs presented a lower biofilm mass without showing differences in the percentage of patients with high counts of Streptococcus mutans and Lactobacilli bacteria [34].

No release of any chemicals that would change the microbiota in the oral cavity were described in those studies [31,32,33,34], but other studies found in the literature review [35] analyzed a cellulose-based thermoformable material used for invisible braces that could be loaded with essential oils (EOs) that have antibacterial and antifungal properties. The loaded materials showed stable mechanical characteristics, allowing them to serve their traditional intended use as clear aligners, nightguards, or retainers. In this context, the integration of antimicrobial essential oils in customized invisible dental aligners could illustrate an efficient approach towards preventive dental care through orthodontics treatments. Overall, in addition to its antimicrobial effects, the loaded cellulose-based thermoformable material presented could provide a valuable platform for drug delivery in a personalized manner directly to the tooth surface and gingiva. We should bear in mind these findings when conducting future research.

Due to the consistency of the results, more studies that include long-term RTCs with large samples, cleaning protocols, and novel techniques, such as those using microbial RNA techniques, should be carried out to determine the reliability of the findings.

### Limitations of the Study

The discussion acknowledged several limitations within the reviewed studies, including small sample sizes, short study durations, and a lack of long-term data. These factors may limit the generalizability of the findings. Furthermore, the variability in study methodologies and the specific populations studied could affect the applicability of the results across different demographic groups. The need for more comprehensive studies featuring diverse patient demographics and longer follow-up periods was highlighted as essential for confirming the long-term benefits of clear aligners on oral health. Additionally, expanding the research to include a variety of age groups and broader geographic regions would help to enhance the understanding of clear aligners’ effectiveness across different populations.

In our systematic review, we have analyzed the existing evidence about periodontal health indicators and oral microbiota. We can consider that only few studies were published on these topics that could be quantitatively synthesized, and a thorough assessment of the overall impact of CA on oral health indicators is therefore difficult. Since orthodontic treatments last over time, analyzing the influence on treatment modalities on oral health indicators should be considered as extremely important. A meta-analysis should be conducted in the future, especially for highlighting the behavior of oral health variables over time. We found a small number of randomized studies with a high-quality design that have been included, the majority being non-randomized. It should be underlined that, although high-quality RCTs are regarded as the gold standard among clinical trials, high-quality cohort studies can also be adequate to study this topic. Moreover, the quality assessment underlined how the most prevalent bias was related to “blinding”, something that is unavoidable due to the nature of the appliance being both FA and CA clearly visible to the participants and the personnel involved in the study. Moreover, when assessing periodontal health, the many indexes and a lack of standardization made it difficult to combine them in a quantitative synthesis.

## 5. Conclusions

Clear aligner systems seemed to positively affect the oral microbial community in the sectors of health and composition, showing a more positive impact on maintaining a balanced oral microbiota when compared to fixed appliances.

## Figures and Tables

**Table 1:** The search strings inserted in the search tool of PubMed, Scopus, and Web of Science. The number of retrieved items (N) are presented in this table.

Database	Search String	N
PubMed	(((“clear”[All Fields] OR “cleared”[All Fields] OR “clearing”[All Fields] OR “clearings”[All Fields] OR “clears”[All Fields]) AND (“align”[All Fields] OR “alignability”[All Fields] OR “alignable”[All Fields] OR “aligned”[All Fields] OR “alignement”[All Fields] OR “aligner”[All Fields] OR “aligners”[All Fields] OR “aligning”[All Fields] OR “alignment”[All Fields] OR “alignments”[All Fields] OR “aligns”[All Fields])) OR (“orthodontic appliances, removable”[MeSH Terms] OR (“orthodontic”[All Fields] AND “appliances”[All Fields] AND “removable”[All Fields]) OR “removable orthodontic appliances”[All Fields] OR “invisalign”[All Fields]) OR (“orthodontic appliances, fixed”[MeSH Terms] OR (“orthodontic”[All Fields] AND “appliances”[All Fields] AND “fixed”[All Fields]) OR “fixed orthodontic appliances”[All Fields] OR (“fixed”[All Fields] AND “appliances”[All Fields]) OR “fixed appliances”[All Fields])) AND (“microbiota”[MeSH Terms] OR “microbiota”[All Fields] OR “microbiotas”[All Fields] OR “microbiota s”[All Fields] OR “microbiotae”[All Fields])	72
Scopus	clear AND aligners OR invisalign OR fixed AND appliances AND microbiota AND PUBYEAR > 2009 AND PUBYEAR < 2024 AND (LIMIT-TO (LANGUAGE, “English”))	62
WOS	(((ALL = (Clear Aligners)) OR ALL = (Invisalign)) OR ALL = (fixed appliances)) AND ALL = (Microbiota)	51

**Table 2:** The key characteristics of the included studies. Abbreviations: clear aligners (CAs); conventional fixed appliances (FAs); aesthetic brackets (CFAs); randomized clinical trial (RCT); males (M); females (F); decay-missing-filled teeth (DMFT), plaque index (PI); gingival index (GI); gingival bleeding index (GBI); sulcus bleeding index (SBI); papilla bleeding index (PBI); bleeding on probing (BOP); basic periodontal exam index (BPE).

Author (Year)	Country	Study Type	Age Years	Assessment Timing	Number of Patients Included/Sex	Outcome Variables	Conclusions
Marincak Vrankova, Z. et al., 2022 [21]	Czech Republic	Pilot study	FAs: 16.8 (14.8)	T0: Before the bonding of fixed orthodontic appliances. T1: during the treatment, by or before the end of the 7th month of the treatment.	FAs: 14 M; 16 F	Decay-missing-filled teeth (DMFT), plaque (PI), and gingival indices (GI).	Deterioration in oral health status, demonstrated by increased plaque index and microbial diversity changes, emphasizing the need for comprehensive oral health management during orthodontic treatment.
Jing, D. et al., 2019 [22]	China	Prospective comparative cohort study	FAs: 16.64 ± 2.03	T1: Prior to bonding. T2: Three months after bonding T3: Six months after bonding. T4: Eighteen months after bonding.	FAs: 5 M; 10 F	Salivary parameters, including secretory immunoglobulin A (sIgA), myeloperoxidase (MPO) and lactate dehydrogenase (LDH), immune response and inflammatory processes.	Significant increase in S. mutans during long-term orthodontic treatment with conventional braces, suggesting a higher risk of white spot lesions compared to self-ligating braces.
Babikow, E. et al., 2023 [23]	USA	Longitudinal observational cohort study	Mean 12.8 ± 1.5 Age Range: 10–15	T0: Just prior to bonding of fixed appliances. T1: 1 week after bonding. T2: Six weeks after bonding. T3: Twelve weeks after bonding.	FAs: 24; 13 F; 11 M	BOP; GI; PDs; PI RNA-sequencing and microbiome bioinformatics analysis from plaque and saliva.	Significant dysbiosis with an increase in disease-associated and decrease in health-associated microbial species due to fixed orthodontic appliances.
Sfondrini, M. et al., 2019 [24]	Italy	Parallel group, randomized, actively controlled trial with a 1:1 allocation ratio.	Trial group (CAs): 33 ± 10.4 Control group (no treatment): 36 ± 9	T0: Fourteen days from the professional hygiene treatment (control group) and before wearing the first clear aligner (trial group). T1: After 2 months from T0.	40 patients: 20 trials CAs; 20 controls (no treatment)	PPD; BOP; PI microbiological tests were performed by means of a real time PCR-based test.	Aligner therapy showed no significant changes in total bacterial load or periodontal pathogens, indicating a stable oral microbiome compared to non-orthodontic patients.
Sanz-Orrio-Soler, I. et al., 2020 [18]	Spain	Prospective controlled trial	FAs and CFAs, mean age of 19.5 years	T0: Prior to starting orthodontic treatment. T1: One month after the start of treatment. T2: Six months after the start of treatment. T3: One year after the start of treatment. T4: Six months after completion of orthodontic treatment.	43 M; 80 F (not indicated the groups)	Sample handling and enumeration of Candida.	Fixed appliances did not significantly affect the presence or level of Candida albicans colonization, challenging assumptions about hygiene and Candida growth under orthodontic conditions.
Levrini, L. et al., 2015 [19]	Italy	RCT	Global age range 16–30 Mean 24.3	T0: Beginning of treatment. T1: Three months into treatment.	CAs: 27 F; 5 M FAs: 17 F; 18 M CG: 8 F; 2 M	PI; BOP; PD periodontal pathogens and microbial biofilm mass through real time PCR.	Patients with CAs exhibited superior periodontal health short-term when compared to those with FAs, suggesting aligners as a preferable option for patients at risk of periodontal disease.
Mulla Issa, F. et al., 2020 [20]	China	Observational Cross-sectional	CAs: 26.85 ± 4.83 FAs: 26.65 ± 5.15 CFAs: 27.65 ± 8.15 SLB: 26.85 ± 5.19	n/s; At least 6 months into treatment.	CAs: 12 F; 8 M FAs: 7 F; 13 M CFAs: 11 F; 9 M SLB: 10 F; 10 M	PI; GI; GBI; SBI; PBI; BOP; BPE.	CAs showed better periodontal indices compared to FAs and CFAs, highlighting the benefits of clear aligners in maintaining oral hygiene and periodontal health.
Kado, I. et al., 2020 [25]	Japan	Prospective controlled trial	FAs: T0: 22.1 ± 8.2 T1: 21.3 ± 7.8 T2: 29.3 ± 12.3	T0: Before placement of FAs. T1: Six months after the start of treatment. T2: Immediately after appliance removal.	FAs: T0: 22 F;6 M T1: 19 F; 7 M T2: 14 F; 3 M	16 S rRNA gene meta-sequencing of supragingival plaque and saliva.	The oral microbiome measured in plaque and saliva changed during orthodontic treatment using fixed appliances. The shift represented an increase in anaerobes and periodontal pathogens and a decrease in commensal bacteria. Specifically, we propose that this shift, particularly in the supragingival microbiome, represents a transition between health and periodontitis.

## Data Availability

The original contributions presented in the study are included in the article, further inquiries can be directed to the corresponding authors. The review protocol was registered in the Open Science Framework ((OFS), https://osf.io/jurxe/ accessed on 24 September 2024), but it is not publicly accessible.

## Supplementary Materials

The following supporting information can be downloaded at: https://www.mdpi.com/article/10.3390/jcm13237435/s1, Table S1: Risk of bias of the included articles using the NOS; Figure S1: Risk of bias of the included RCTs according to the Cochrane Rob-1 tool. Low Risk (Green); Unclear (Yellow); High Risk (Red); Figure S2: Flow chart.
