# Supplementary material for: Changes in the Oral Microbiota with the Use of Aligners vs. Braces: A Systematic Review
Source: J Clin Med. 2024 Dec 6;13(23):7435. doi: 10.3390/jcm13237435 (PMC11642593; doi:10.3390/jcm13237435)

TABLES

Table S1: Risk of bias of the included articles using the NOS (cohort studies)

			Babikow et al. 2023	Jing et al. 2019	Kado et al. 2020	Marincak Vrankova et al. 2022	Mulla Issa et al. 2020	Sanz-Orrio-Soler et al. 2020	Sfondrini et al. 2019
SELECTION	Representativeness of the exposed cohort	truly representative of the average	❋	❋	❋	❋	❋	❋	❋
		somewhat representative of the average							
		selected group of users							
		no description of the derivation of the cohort							
	Selection of the non-exposed cohort	drawn from the same community as the exposed cohort	❋	❋	❋	❋	❋	❋	❋
		drawn from a different source							
		no description of the derivation of the non-exposed cohort							
	Ascertainment of exposure	secure record	❋	❋	❋	❋	❋	❋	❋
		structured interview							
		written self-report							
		no description							
	Demonstration that outcome of interest was not present at start of study	yes	❋	❋	❋	❋	❋	❋	❋
		no							
COMPARA-TIBILITY	Comparability of cohorts on the basis of the design	study controls					❋	❋	❋

	or analysis								
		study controls for any additional factor	✵	✵	✵	✵			
	Assessment of outcome	independent blind assessment							
		record linkage	✵	✵	✵	✵	✵	✵	✵
		self-report							
		no description							
OUTCOME	Was follow-up long enough for outcomes to occur	yes	✵	✵	✵	✵	✵	✵	✵
		no							
	Adequacy of follow up of cohorts	complete follow up - all subjects accounted for	✵	✵	✵	✵	✵	✵	✵
		subjects lost to follow up unlikely to introduce bias							
		no description of those lost							
		no statement							

FIGURES

Figure S1: Risk of bias of the included RCTs according to the Cochrane Rob-1 tool. Low Risk (Green); Unclear (Yellow); High Risk (Red).

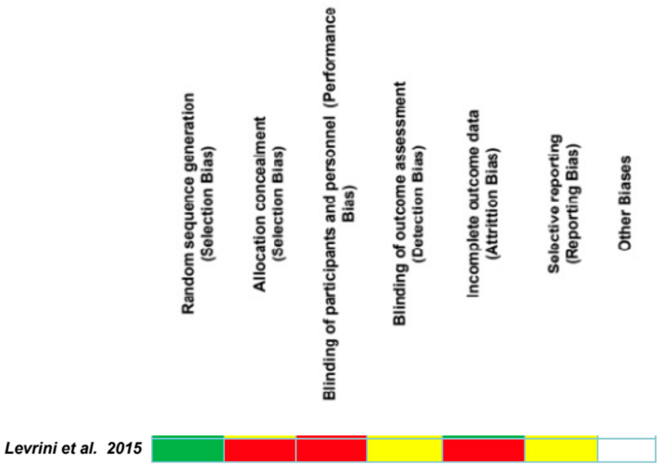

Figure S2: Flow chart

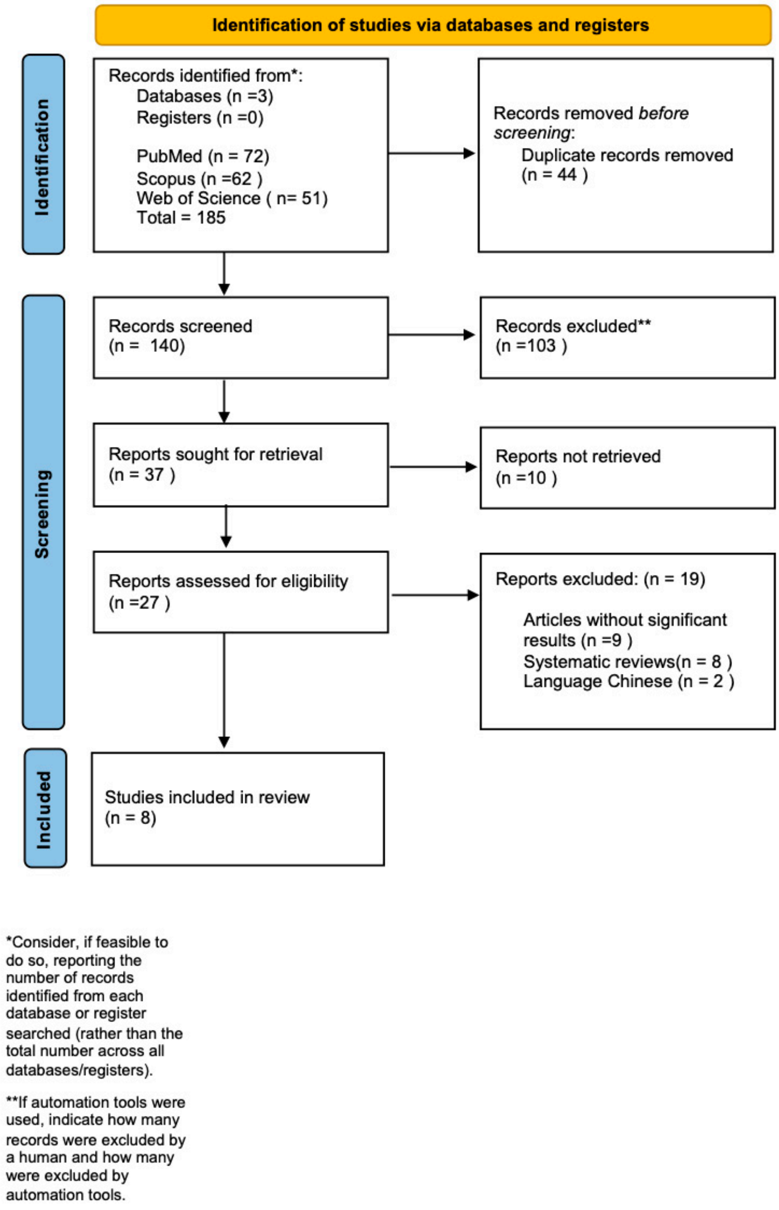

Supplement: Supplementary file 1 [file jcm-13-07435-s001.zip › jcm-3261995-supplementary.pdf]
